# 3D fluorescent in situ hybridization using Arabidopsis leaf cryosections and isolated nuclei

**DOI:** 10.1186/1746-4811-5-11

**Published:** 2009-08-03

**Authors:** Leïla Tirichine, Philippe Andrey, Eric Biot, Yves Maurin, Valérie Gaudin

**Affiliations:** 1 Laboratoire de Biologie Cellulaire, INRA UR 501, IJPB, Route de Saint-Cyr, F-78026 Versailles, France; 2 Neurobiologie de l'Olfaction et de la Prise Alimentaire, INRA UMR 1197, Domaine de Vilvert, F-78350 Jouy-en-Josas, France; 3 Université Paris-Sud 11, UMR 1197, F-91400 Orsay, France; 4 IFR 144 Neuro-Sud, Paris, France; 5 Université Pierre et Marie Curie, Paris, France; 6 Institut des Sciences du Végétal, CNRS, avenue de la Terrasse, F-91198 Gif-sur-Yvette, France

## Abstract

**Background:**

Fluorescent hybridization techniques are widely used to study the functional organization of different compartments within the mammalian nucleus. However, few examples of such studies are known in the plant kingdom. Indeed, preservation of nuclei 3D structure, which is required for nuclear organization studies, is difficult to fulfill.

**Results:**

We report a rapid protocol for fluorescent *in situ *hybridization (FISH) performed on 3D isolated nuclei and thin cryosectioned leaves of *Arabidopsis thaliana*. The use of direct labeling minimized treatment steps, shortening the overall procedure. Using image analysis, we measured different parameters related to nucleus morphology and overall 3D structure.

**Conclusion:**

Our work describes a 3D-FISH protocol that preserves the 3D structure of *Arabidopsis *interphase nuclei. Moreover, we report for the first time FISH using cryosections of *Arabidopsis *leaves. This protocol is a valuable tool to investigate nuclear architecture and chromatin organization.

## Background

The organization of chromatin within the cell nucleus has important implications in gene expression. Several studies have aimed to better understand how gene regulation is organized within chromosome territories and how it relates to various level of chromatin folding [1,2]. Defining the underlying mechanisms responsible for the positioning and dynamics of various nuclear entities within the nuclear space is a major challenge because it requires the development of specific three-dimensional (3D) techniques preserving nuclear integrity and spatial organization.

In mammals, fluorescent *in situ *hybridization of nuclei with a three-dimensionally preserved organization (3D-FISH) has proven to be a useful tool for such studies [3-7]. In plants, 2D-FISH has been widely used to better understand the organization of nuclear components [8,9]. Recently, plant 3D-FISH protocols using whole or vibratome-sectioned specimens have been developed [10-14]. The most challenging issue for 3D-FISH studies is to preserve the morphology and the structure of the cell nuclei and to minimize nuclear and chromatin modifications during hybridization. Methanol and acetic acid fixation procedures are efficient treatments to eliminate the cytoplasm and thus allow easier penetration of DNA probes. Although widely used in 2D-FISH, these fixative reagents are not suitable for 3D-FISH because they induce nuclear modifications [15]. Furthermore, harsh chemical treatments such as ethanol can alter chromatin organization [16].

Here, we report an alternative protocol for investigating nuclear organization in *Arabidopsis *nuclei and cryosectioned leaf tissue. To preserve chromatin structure and nuclear morphology, we avoided treatments such as enzymatic digestion, ethanol and heat-dehydration and we reduced thermal denaturation treatments. Using direct labeling, we minimized post-hybridization steps, shortening the procedure and allowing the protocol to be carried out within two days. DAPI counterstaining and digital image analysis assessing nuclear morphological parameters such as volume, compactness and flatness, indicated that this protocol effectively preserves 3D nuclear structure.

### Materials

#### Plant material

*Arabidopsis thaliana *accession Columbia (Col-0) seeds were surface sterilized and grown *in vitro *at 20°C, 75% humidity, 60 μmol m^-2 ^s^-1 ^and under a 10 hours light/14 hours dark period as described previously [17].

#### DNA probes

We used the following DNA clones: 180 bp pAL1 [15], 5S rDNA pCT4.2 [16], 45S rDNA [17], Bacterial Artificial Chromosomes (BAC) T6P5 (GenBank Accession AC005970) and T1J1 (GenBank Accession AF128393), located on small arm of chromosome 2 and 4, respectively. T6P5 and T1J1 belong to the tiling paths of BAC clones selected for chromosome painting: they contain <5% of mobile elements and are specific to chromosome 2 and 4 [18]. DNA was extracted using Nucleobond extraction kit (Clontech) according to the manufacturer's instructions.

#### Reagents and buffers

Triton X-100.

Dextran sulfate 20%.

RNAse (Sigma AR-6513, stock solution 10 mg/ml).

Klenow fragment (40 U/μl) (Invitrogen Y01396).

BioPrime^® ^DNA Labelling System (Invitrogen 18094-011: 2.5× random primers solution 125 mM Tris-HCl (pH 6.8), 12.5 mM MgCl_2_, 25 mM β-mercaptoethanol, 750 μg/ml oligodeoxyribonucleotide primers (random octamers) (Invitrogen Y01393).

Fluorochromes: ChromaTide^® ^Alexa Fluor^® ^488-5-dUTP (Invitrogen C11397), Cy3-dUTP (Amersham PA53022), Cy5-dUTP (Amersham PA55022). Prepare a 10× fluorophore mixture (1 mM dATP, 1 mM dCTP, 1 mM dGTP, 0.65 mM dTTP, 0.35 mM fluorochrome).

DAPI (4'6-Diamidino-2-phenylindole) at 1 μg/ml final concentration into VECTASHIELD^®^ antifade mounting medium (Vector Laboratories).

Cryomount medium (Histolab 00890).

Nuclei extraction buffer (10 mM Tris HCl pH 7, 4 mM spermidine, 1 mM spermine, 5 mM MgCl_2_, 0.1% triton X-100).

1× Phosphate-Buffered Saline (PBS) pH 7.3 (3.2 mM Na_2_HPO_4_, 0.5 mM KH_2_PO_4_, 135 mM NaCl, 1.3 mM KCl).

20× SSC (3 M NaCl, 0.3 M sodium citrate, pH 7.0).

Stop buffer 0.5 M Na_2_EDTA pH 8.0 (Invitrogen 50690).

HB50 (50% deionized formamide, 2× SSC, 50 mM phosphate, pH 7.0).

#### Equipment

Vacuum pump.

Centrifuge Sigma 3K18 rotor 11133.

Nanodrop, Labtech spectrophotometer ND-1000.

Hot plate (75–80°C) and heating block (100°C).

Incubators (37°C and 55°C) and a moist chamber.

Coplin jars.

Silanized slides (Dako S3003) or SuperFrost^® ^Plus glass slides (Menzel-Gläser J1800AMNZ).

Coverslips (22 × 22 and 24 × 50 mm).

Dako pen (Dako S2002).

Cryostat (Leica CM-3050S).

### FISH Protocol

#### Fixation

Fix young seedlings (18 days-post-germination) in 4% paraformaldehyde (PFA) in 1× PBS buffer at room temperature (RT) for 30 minutes under a vacuum. Replace the fixative solution with fresh fixative solution and fix for an additional 30 minutes under a vacuum. Rinse seedlings in 1× PBS twice for 5 minutes each and store in 1× PBS at 4°C until use (fixed seedlings can be stored for up to a month).

#### Nuclei extraction

The extraction protocol is based on the existing protocol reported by [19]. All centrifugation steps were carried out at 500 g (1700 rpm) for 3 min, at room temperature.

Place up to 8 seedlings in a 1.5 ml Eppendorf tube containing 500 μl of nuclei extraction buffer. Prior to extraction, add β-mercaptoethanol to a final concentration of 5 mM. Extract nuclei gently with a plastic pestle. Filter the nuclei suspension through a 50 μm nylon mesh. Discard the supernatant after centrifugation and wash the pellet with 1× PBS. Treat the pellet with 300 μl of 1× PBS containing 0.5% triton X-100 for 5 minutes. Centrifuge, discard the supernatant and wash the pellet with 1× PBS for 3 minutes.

Resuspend the pellet in 30 μl of 1× PBS. Add 4 μl of this suspension to a slide and dry the slide at 4°C for 20 minutes before mounting with DAPI or carrying on with the FISH procedure. Draw a circle around the drop of nuclei on the slide using a DAKO pen to avoid deforming the specimen once mounted with a coverslip.

#### Cryostat sectioning

To prepare tissue for cryostat sectioning, cut leaves into small pieces (about 5 mm^2^) and incubate the leaf sections in 15% sucrose overnight at 4°C. Place a piece of leaf on a slide, remove the excess of sucrose and add to the leaf a drop of mounting medium. Transfer the slide to a cryostat chamber maintained at -25°C during sectioning and leave the slide within the cryostat chamber until the mounting medium and leaf sample are frozen. Remove the frozen sample from the slide with forceps and transfer it to a mounting stub in an upright position for transversal sectioning. Add few μl of mounting medium and correct the orientation of the sample being careful to maintain it in an upright position until the mounting medium freezes.

To section the tissue, transfer the sample to the holder and adjust the orientation of the sample, the microtome knife and the anti-roll plate. For FISH studies, we used 10, 16 and 20 μm sections. After sectioning, carefully place the leaf sections on a SuperFrost or silanized slide that has been pre-cooled to -25°C. The sample can be stored on the slide at -20°C until use. Let the slide thaw at room temperature before washing in 1× PBS for 5 minutes to remove the mounting medium.

#### Pretreatments and prehybridization

Incubate leaf sections with 1× PBS containing 0.5% triton X-100 for 10 minutes or incubate isolated nuclei with 1× PBS containing 0.5% triton for 3 minutes at RT. Wash the slide with 1× PBS for 5 minutes to remove the detergent and proceed to the HCl treatment. Treat leaf sections with sterile water containing 0.1 N HCl for 20 minutes at RT. Rinse the slide with sterile water for 5 minutes and proceed directly to the RNase treatment. For the RNase treatment, equilibrate the slide in 2× SSC for 5 minutes at RT then incubate the slide in 2× SSC containing 400 μg/μl RNase for 15 minutes in a moist chamber at 37°C. Rinse the slide in 2× SSC for 2 minutes at RT. Apply the HB50 prehybridization mixture (~100 μl) to the slide, place a coverslip over the sample and incubate it in a moist chamber at 37°C for 1 to 2 hours.

#### Random primer probe labeling

On ice, add 20 μl of 2.5× random primers solution to 20 μl DNA (100 ng). Denature this mix at 100°C for 10 minutes and immediately cool it on ice. Add 5 μl of fluorochrome mixture and distilled water to a total volume of 49 μl. After a brief vortexing, add 1 μl of klenow enzyme. Mix gently by pipetting up and down and centrifuge the reaction for 30 seconds. Incubate the reaction at 37°C for 1 hour to yield 100 to 200 base pairs (bp) fragments. Load 5 μl of the reaction on a 1% agarose gel to verify the length of the DNA fragments. If the DNA length is not between 100 and 200 bp, incubate for an additional hour or up to 24 hours. Stop the reaction, by adding 5 μl of stop buffer and store the probe at -20°C until use.

#### Hybridization

Prepare the probe mix as follows: 10 μl of 20% dextran sulfate, 1 to 9 μl HB50 (50% deionized formamide in 2× SSC and 50 mM sodium phosphate, pH 7.0), 1 to 9 μl of probe and adjust to a final volume of 20 μl. Use 100 to 400 ng of DNA for BAC clones and 20 to 100 ng of DNA for repeated DNA. Denature the probe at 100°C for 5 minutes and transfer immediately to ice.

Denature the sample at 75°C for 5 minutes, drain from the denatured slide any excess fluid, and add 20 μl of denatured probe to the sample. Cover the sample with a coverslip and hybridize it in a moist chamber at 37°C for 2 to 3 hours or overnight for convenience.

#### Post-hybridization washes and detection

Wash sample as follows: 2 washes in 2× SSC for 15 minutes each at 55°C; 1 wash in 1× SSC for 10 minutes at 55°C and 1 wash in sterile water for 2 minutes at RT. Mount the slide in 3 μl of Vectashield containing 1 μg/ml DAPI. Seal the coverslip with colorless nail polish and store at 4°C (sealed samples can be stored up to a month at 4°C).

#### Microscopy and image acquisition

All images were taken with a Leica DMIRE2 SP2 confocal microscope (Leica Microsystems, Heidelberg, Germany). A UV diode (405 nm) was used for DAPI visualization and three lasers at 488 nm, 543 nm and 633 nm were used for the excitation of Alexa Fluor^® ^488-5-dUTP, Cy3-dUTP and Cy5-dUTP, respectively. Images were taken through a 63× water immersion objective (NA 1.2, WD 0.2 mm). Z series images were collected at 0.120 μm intervals. Images were scanned with a zoom of 9.29, a frame average of 2 and a line average of 2.

### Image processing and analysis

To investigate the impact of the FISH treatments on nucleus morphology, confocal images of DAPI stained nuclei were acquired before and after FISH. Leaf nuclei of untreated cryosections were used as a reference. After automatic extraction of nucleus masks from the confocal images, morphometrical parameters of the three nucleus classes were extracted and compared.

#### Nucleus image segmentation

Confocal image stacks were automatically converted to a format enabling their processing by programs developed using the Free-D software libraries [20]. To separate the nucleus from the background, a preliminary intensity threshold was computed using the isodata algorithm [21]. Since this algorithm is sensitive to the relative size of the nucleus within the image, the threshold was generally too high because of the larger background size. This bias was corrected by setting the actual threshold to m-2s, where m and s are the intensity average and standard-deviation, respectively, computed over the nucleus region defined by the preliminary threshold. Holes due mainly to the nucleolus, boundary irregularities due to noise, and bumps due to blur from chromocenters [22] were smoothed out using hole filling, opening, and closing binary morphological operators [23]. Finally, a surface model of the nuclear envelope was generated by applying the marching cubes algorithm [24] to the nucleus binary mask.

#### Nucleus morphological analysis

Nucleus size was quantified from the equivalent spherical diameter, i.e., the diameter of the sphere with the same volume as that enclosed by the nuclear surface. Nucleus global shape was quantified using the compactness parameter: Compactness = 36 π volume^2^/surface area^3^.

This parameter, which is comprised between 0 and 1, characterizes shape regularity and takes its maximum value for a sphere. To quantify an eventual flattening of the nucleus, a flatness parameter was derived from the lengths of the principal axes of the nuclear surface: Flatness = length of intermediate axis/length of shortest axis. Flatness was only computed over the nuclei for which the shortest axis corresponded to the Z (optical axis) direction.

Morphological parameters measured on isolated nuclei before or after FISH were compared to measures made on nuclei from leaf sections with the Mann-Whitney test (alpha = 5%) using the R statistical software [25].

## Discussion

Different parameters were chosen and tested to set up the FISH protocol on isolated nuclei and leaf cryosections. Paraformaldehyde fixative was selected over other fixatives because it was previously demonstrated to better preserve chromatin structure and nuclear morphology [26-28]. The fixation time was optimized to find a compromise between the preservation of the morphology and the extensive cross-linking of the proteins surrounding the DNA. Indeed cross-linking of chromatin complexes masks the availability of the target DNA to labeled probes, requiring harsh permeabilization procedures that could compromise the preservation of nuclei 3D structure for hybridization.

Triton X-100 combined with saponin and additional treatments such as repeated freeze thawing in liquid nitrogen were previously used to permeabilize human and mouse cell nuclei [4,26,29,30]. In these studies, an overall conservation of nuclear morphology and chromatin structure was reported. Based on these previous studies, we tested different detergents, triton X-100, saponin and lipsol, individually and combined, at various times and concentrations. Triton X-100 allowed an efficient lysis of chloroplasts and maximized the removal of cytoplasm from both sections and isolated nuclei. With triton X-100, we obtained good quality FISH signals whereas with saponin and lipsol the nuclei were poorly preserved and we obtained weak signals or no signal.

Specimen treatment by HCl is widely used in FISH protocols for animal and plant cells and tissues [29-31]. It favors probe penetration, thus improving the signal to noise ratio. We observed brighter FISH signals from HCl-treated leaf cryosections than from untreated cryosections, which either yielded faint signals or no signal.

Indirect probe labeling is the most commonly used labeling in plant FISH protocols [11,12,17]. We chose direct labeling, since it shortened the procedure and led to to a better sample preservation. Indeed the samples went through fewer steps for signal detection (eg. no incubation with primary and fluorescent conjugated antibodies and no subsequent washes). Furthermore, compared to nick translation, random primers labeling yielded higher incorporation rates of the fluorochrome and generated smaller size fragments, favoring probe penetration [32].

Most protocols published thus far use either 80°C or higher temperatures for denaturation (e. g. 85°C, 94°C)[4,13,33]. In this study, thermal denaturation of *Arabidopsis *nuclei was performed at 70°C, 75°C, 88°C and 94°C in 50% formamide hybridization buffer. After FISH, DAPI staining was performed and nuclei were analyzed. A denaturation temperature of 75°C was retained for the protocol because at 75°C the nuclear structure with the typical compact and well-defined centromeres was maintained and a high quality hybridization signal was observed.

**Figure 1:**
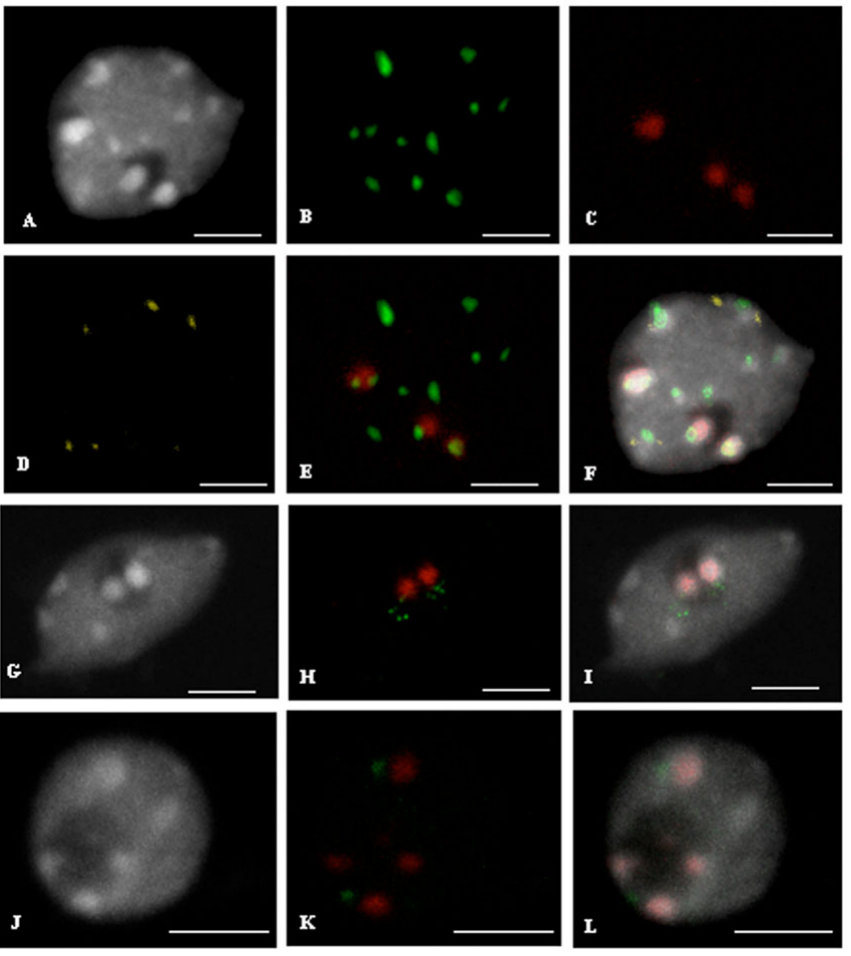
**FISH on isolated interphase nuclei**. (A, G, J) DAPI staining. (B) centromeric 180 bp repeats (Alexa Fluor^® ^488-5-dUT, green). (C) 45S rDNA (Cy5-dUTP, red). (D) 5S rDNA (Cy3-dUTP, yellow) (E) Merge with 180 bp repeats and 45S rDNA visible as green and red, respectively. (F) Merge with DAPI, 180 bp repeats, 45S rDNA and 5S rDNA. Bar = 2 μm. (H) FISH with 45S rDNA and T1J1 BAC specific of chromosome 4, visible as red and green signals, respectively. (I) Merge. Bar = 4 μm. (J, K, L) FISH on an isolated interphase nucleus. (J) DAPI. (K) 45S rDNA and BAC T6P5 specific of chromosome 2, visible as red and green, respectively. (L) Merge. Bar = 2 μm. All images are maximum projections of confocal image stacks.

**Figure 2:**
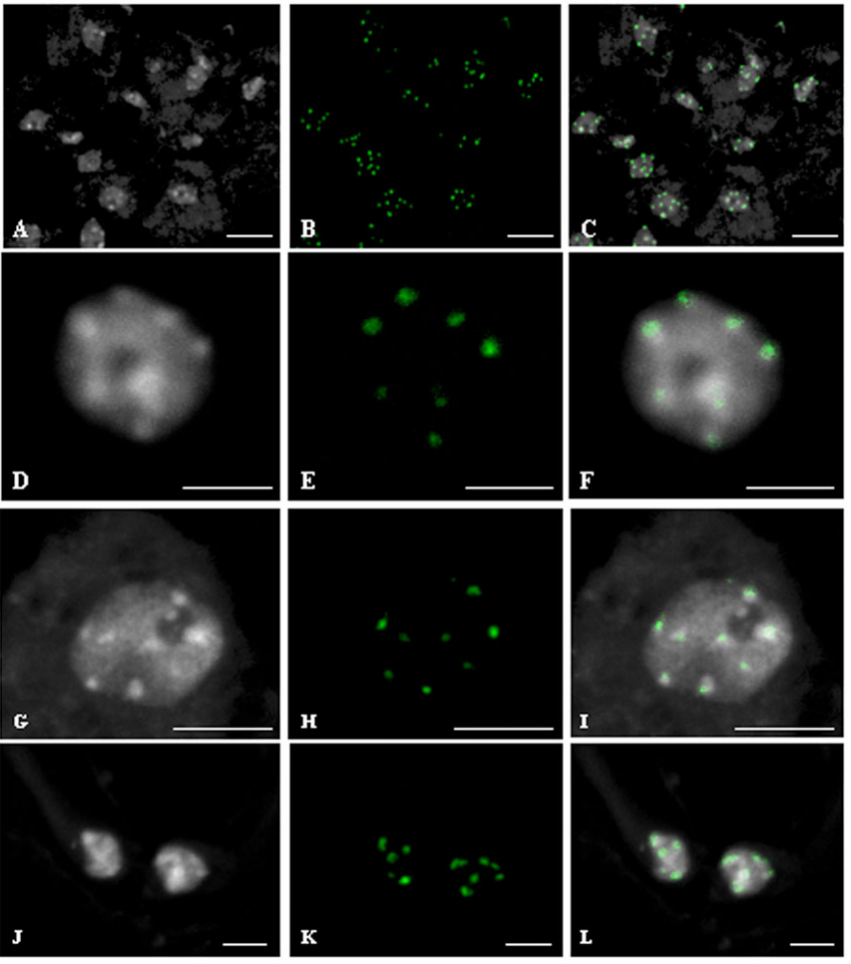
**FISH localization of centromeric 180 bp repeats on 20 μm cryosections**. (A, D, G, J) DAPI counterstaining. (B, E, H, K) localization of 180 bp repeats. (C, F, I, L) merged images. (A, B, C) cryosection. Bar = 10 μm. (D, E, F) Epidermis nucleus. Bar = 5 μm. (G, H, I) Subepidermis nucleus. Bar = 5 μm. (J, K, L). Guard cell nuclei. Bar = 2 μm.

Representative FISH signals from isolated nuclei labeled with repetitive sequences or BACs specific for chromosomes 2 and 4 are presented in Figure 1. To retain nuclei within the context of their intact tissue environment and keep information on their cellular identity, we developed FISH on leaf cryosections, choosing centromeric repetitive sequences to set up the technique (Figure 2). This technique has the advantage over other embedding techniques using paraffin or resin to be possibly coupled with FISH without dehydration procedures. Indeed, dehydration procedures were shown to negatively affect nuclei morphology [16]. Our FISH protocol combines preservation of nuclear architecture and high resolution imaging of cell nuclei.

Three morphological parameters were used to investigate whether the protocol preserves the overall 3D structure of nuclei, using DAPI stained nuclei from leaf cryosections that have not been FISH treated as reference nuclei. Indeed, because the cell nuclei are embedded within the tissues, their morphology likely closely reflects their *in vivo *morphology. Table 1 compares the morphological parameters measured on isolated nuclei before and after FISH procedures to reference nuclei from untreated leaf cryosections. No difference in either size or shape, as quantified by the equivalent spherical diameter and compactness, respectively, was noted. In all three groups, most of the nuclei had their shortest axis close to the Z direction (see column NZ). However, there was no flatness difference, either before or after FISH, with the reference nuclei. Overall, this quantitative morphological analysis strongly suggests that the 3D-FISH protocol preserves nucleus size and shape.

**Table 1:** Morphological parameters of isolated nuclei before and after FISH treatments compared to untreated nuclei from leaf cryosections.

	N	Diameter (μm)	Compactness	NZ	Flatness
Untreated	65	5.99 ± 0.72	0.79 ± 0.07	21	1.54 ± 0.36

Before FISH	77	5.85 ± 0.85 (p = 0.25)	0.77 ± 0.11 (p = 0.72)	71	1.56 ± 0.40 (p = 0.87)

After FISH	64	5.79 ± 0.83 (p = 0.14)	0.74 ± 0.14 (p = 0.36)	48	1.79 ± 0.53 (p = 0.08)

N: number of analyzed nuclei; NZ: number of nuclei with the shortest axis aligned with the Z-axis and used to compute flatness. The p-values of the pair wise comparisons between isolated nuclei and untreated cryosection nuclei are given in parenthesis. ±: Standard error. Untreated: cryosections.

## Conclusion

Although 3D-FISH protocols are widely used to characterize nuclear architecture and dynamics in conjunction with changes in gene expression in animal models, few studies have been performed in plants. In the present work, we report a protocol for the isolation of 3D preserved nuclei and cryosections of *Arabidopsis *tissues coupled with a direct labeling FISH procedure. Measurement of morphological parameters and statistical analyses indicated that the 3D nuclear morphology was preserved after the FISH procedure, thus validating the protocol. To our knowledge, cryosectioning of *Arabidopsis *tissues was not previously coupled with FISH and therefore this protocol provides a valuable alternative to other 3D-FISH protocols based on vibratome-sectioned or whole mount specimens to study cell nuclei with defined cell identities [10,12].

## Competing interests

The authors declare that they have no competing interests.

## Authors' contributions

LT and VG designed the experiments. LT did the experiments and drafted the manuscript. PA and EB did confocal images processing and analyses. All authors contributed to the writing and approved the manuscript.
